# Random Access Using Deep Reinforcement Learning in Dense Mobile Networks

**DOI:** 10.3390/s21093210

**Published:** 2021-05-05

**Authors:** Yared Zerihun Bekele, Young-June Choi

**Affiliations:** 1 Department of Artificial Intelligence, Ajou University, Suwon 16499, Korea; yaredzerihun@ajou.ac.kr; 2 Department of Software and Computer Engineering, Ajou University, Suwon 16499, Korea

**Keywords:** machine learning, optimization, random access

## Abstract

5G and Beyond 5G mobile networks use several high-frequency spectrum bands such as the millimeter-wave (mmWave) bands to alleviate the problem of bandwidth scarcity. However high-frequency bands do not cover larger distances. The coverage problem is addressed by using a heterogeneous network which comprises numerous small and macrocells, defined by transmission and reception points (TRxPs). For such a network, random access is considered a challenging function in which users attempt to select an efficient TRxP by random access within a given time. Ideally, an efficient TRxP is less congested, minimizing delays in users’ random access. However, owing to the nature of random access, it is not feasible to deploy a centralized controller estimating the congestion level of each cell and deliver this information back to users during random access. To solve this problem, we establish an optimization problem and employ a reinforcement-learning-based scheme. The proposed scheme estimates congestion of TRxPs in service and selects the optimal access point. Mathematically, this approach is beneficial in approximating and minimizing a random access delay function. Through simulation, we demonstrate that our proposed deep learning-based algorithm improves performance on random access. Notably, the average access delay is improved by 58.89% from the original 3GPP algorithm, and the probability of successful access also improved.

## 1. Introduction

**Figure 1:**
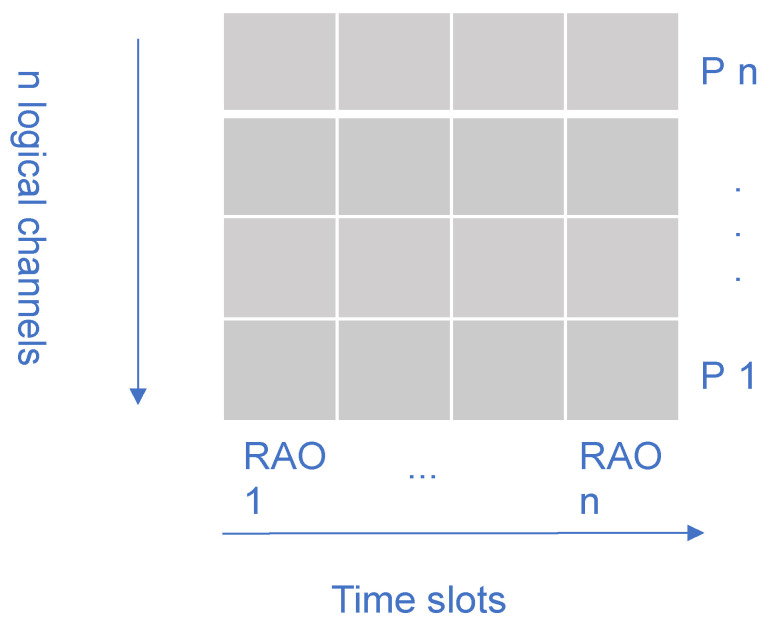
The frame structure of RA typically employed in mobile networks. Preambles ($P_{1}$ up to $P_{n}$) are rotated in every RAO.

In 5G and Beyond 5G cellular networks, random access (RA) protocols maneuver multiple users to negotiate over a small portion of bandwidth before they assent to transmit data on a radio resource. In RA protocols, TRxPs send preambles at the start of the network. Users randomly choose one out of the preambles for negotiating with the TRxP. A preamble is a radio resources for RA consisting of both time and frequency resources and appears in each random access opportunity (RAO) periodically broadcast by TRxPs (i.e., base stations), as depicted in Figure 1 [1]. When users need to connect to the network, they randomly select one out of a set of possible preambles in a given RAO. In the case that two or more users select the same preamble, a collision may occur, and a back-off procedure is initiated. The process repeats until the users succeed in their access attempt, or the network is unreachable after the maximum number of retrials.

RA resources can be requested and re-requested by users under different cases, such as accessing the network for the first time, the loss of system information, change of attachment from the current access point to another due to mobility, etc. [1,2,3,4]. In LTE systems, RA has a long four-step procedure. These systems are exposed to the RA delay issue, which is not beneficial in terms of meeting the stringent latency requirement of 5G mobile network use cases, such as ultra-reliable and low-latency communications (URLLC) services. The delay issue in RA becomes more serious for vehicle-to-everything (V2X) communications, where highly mobile users or vehicles cross a network of dense millimeter-wave (mmWave) TRxPs deployed to relieve a coverage problem.

**Figure 2:**
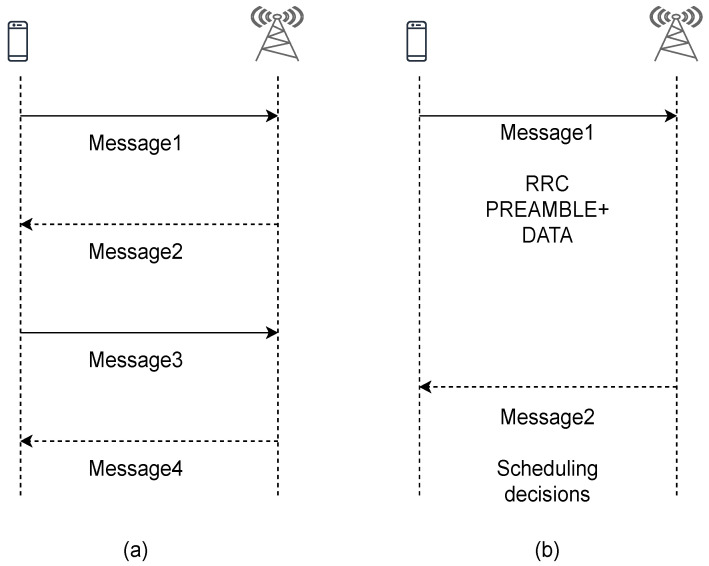
Procedure of RA: (**a**) LTE; (**b**) 5G NR.

Recently, a two-step procedure has been proposed to reduce the RA delay, where a request for RA is transmitted in the first step, and the second step is completed with a scheduling decision, as illustrated in Figure 2. This is applicable to 5G New Radio (NR). However, the search for protocols and algorithms that further reduce the RA delay remains an area of active research. In this paper, we focus on the most significant factor contributing to RA delay, which is the congestion that occurs at the TRxPs due to the influx of 5G and Beyond 5G traffic. Mobile nodes are associated with TRxPs frequently and near-instantaneously, thus triggering RA consecutively. As a result, some TRxPs may become congested by excessive requests from too many users, even if they have enough RA preambles to accommodate users in a given RAO.

Because these networks include many heterogeneous cells (small and macro-cells), users can leverage this feature and intelligently choose a TRxP by estimating the random access delay caused by congestion. A smart user application able to estimate the current congestion level of a TRxP before connecting to it is expected to reduce delay and reduce congestion. Previous methodologies reach maximum signal-to-noise ratios for network access, or implement an access class barring (ACB) scheme for machine-type communications [5,6].

We consider mathematical optimization of the RA delay. One must assume a model representation of the network environment to proceed with RA; this is a strong assumption. For example, to minimize the RA delay, an exact analytical expression that captures the context of the communication system should be given. The RA delay is a stochastic variable with options for consistent approximations. However, machine learning techniques are relatively new approaches still being researched for the optimization of such network procedures, which do not require an exact postulation of a complex mathematical function, but rather stimulate an approximation. Therefore, a machine learning approach to find less congested access points helps users learn over time from experience and try to develop a decision strategy (policy) without requiring the strong assumptions for RA delay [7,8,9,10,11,12]. In this paper, we test how reinforcement learning (RL) strategies allow for optimization of the RA procedure when the network model is fully represented as a Markov decision process (MDP).

The main reason for experimenting with reinforcement learning is that transition probabilities are not known in advance in the MDP model described in this paper, due to the lack of a labeled data-set. The partial model of the random access environment with no predefined labeled data-set is such that our agents (users) observe the status of the network, take actions by selecting random access points, and collect rewards and punishments in the form of the environment’s key performance indicators. For value approximation over finitely many states (i.e., many-dimensional input), powerful neural network approximations, as well as deep RL, have recently been proposed to solve the convergence problem [13,14,15].

In light of this, to further reduce the RA delay of users and to overcome the current inefficiency of TRxP selection, we propose a novel deep RL technique where users learn over time, through training, to select an efficient TRxP among available covering access points (TRxPs), then satisfy their RA performance expectations. In particular, the main contributions of this paper are outlined as follows:

- Formulate the random access selection task as a mathematical optimization formulation.
- Define the random access selection task in terms of MDP.
- Propose a novel deep reinforcement learning algorithm that solves the random access selection problem formulated as MDP. This is performed by designing the system states, defining actions that are taken by agents, and defining a reward function.
- Test and compare the performance of the proposed deep reinforcement learning algorithm against another learning algorithm and baseline approaches.

The remainder of this paper is organized as follows. In Section 2, we assess relevant and up-to-date enhancements on RA performance proposed for delay-sensitive use cases of 5G and Beyond 5G (B5G) networks. We discuss the system model and problem formulation in Section 3. Section 4 presents an analysis of our proposed approach and model in terms of feasibility. In Section 5, we present experimental results by simulation. Finally, Section 6 concludes the paper.

## 2. AI and Recent RA Enhancements in Literature

In this section, we discuss literature regarding artificial intelligence. We also revisit some recent proposals for RA enhancement. These recent advancements mainly address some architectural modifications of the procedure and are also specific to each B5G use case.

### 2.1. AI for Wireless Networking

Artificial Intelligence (AI) algorithms act in a way the human mind functions. Cognition and learning are simulated into machines (agents) that interact with their environment to learn meaningful experiences. This is in contrast to the conventional computation paradigms, where algorithms are given a specific set of instructions to operate on inputs to give an output. In AI, however, algorithms start to function from the output and work towards finding the implicit patterns that resulted in the outputs. AI has seen a lot of interesting results in different research domains. The wireless domain is no exception to this.

A survey of AI techniques is given in [16], where authors discuss how AI can be leveraged to improve the design and operation of future generation wireless networks including 5G. It is discussed that the problems found in the design of these networks are unstructured, and hence AI techniques can be helpful. Some discussed techniques are divided according to the problems found in each layer of the protocol stack. A more detailed discussion and survey about these techniques, however, is found in [17]. As an example of such techniques, [18] proposed a fast machine learning algorithm by modeling the problem as a contextual multi-armed bandit one. Authors in [19] proposed a combination of Support Vector Machine(SVM) and Deep Belief Network(DBN) to solve a joint cross-layered problem: scheduling and power allocation.

In particular, for deep learning, a subset of AI [20], one can find its application in all layers of the protocol stack including network security (intrusion detection systems) [21]. Deep learning models allow learning from the data they are trained upon. However, in wireless tasks where an agent has to actively interact with the environment and a reward/punishment signal is available, reinforcement learning can be applied. In the recent advancement in the field of AI, deep reinforcement learning is proposed where deep learning is combined with reinforcement learning. The surveys given in [22,23] discusses deep reinforcement learning applications for wireless networks. For instance, Ref. [24] applied deep reinforcement learning for the resource allocation problem.

### 2.2. Information Redundancy for RA

3GPP first introduced the concept of redundant preambles for narrowband (NB)-IoT, and the authors in [25] tested the feasibility of the proposal in improving initial access probability for 5G mmWave networks for a massive V2X use case, where a massive number of sensors were deployed on vehicles, and subsequently, massive machine-type communications (mMTC) are no longer commonly spatially static. In practice, mMTC traffic tends to flow in bursts, and beam alignment problems along with the harsh propagation conditions of mmWaves are huge challenges to the reliability of the RA process.

Redundant preamble transmission aims to quickly acquire data transmission opportunities in addition to improving the reliability (successful access probability). The mmWave base stations (eNodeBs, gNodeBs, and access points) send variable j in addition to ACB and uniform back-off window (UBW) variables. Here j denotes the number of times a user is allowed to send a selected preamble after selecting o from the set of preambles available. However, the major concern is to dynamically allocate j based on the traffic load. For this, the authors in [26] adopted a previously developed algorithm calculating an optimal j by solving an optimization problem.

Other studies in [27,28] considered an information redundancy approach in which RA response (RAR) messages are redundantly sent to users to reduce the collision rate and hence support an envisioned a large number of users. In the legacy system, if more than one user gets the same preamble, they collide or one of them obtains a RAR response. Owing to the redundant RAR responses, a user randomly selects a single RAR message as they are different from each other and has a chance to proceed further in the procedure. They experimentally demonstrated that the performance of RA increases as the number of redundant RAR messages increases. This tends to occur when user density is very high. 5G NR adopted the legacy RA procedure from LTE; however, a beam selection enhancement was added. Users are expected to synchronize with a selected beam to perform the procedure.

The authors in [29,30,31,32,33] suggested an interesting analysis that can be applied to various RA processes. Users participating in the RA procedure are considered analogous to stochastic experiments involving the computation of a probability distribution and expectations of some of the random variables involved in the analysis. The random variables are the control parameters discussed in this paper. Assuming the RA channel (RACH) as a queueing system, probabilistic upper bounds for performance metrics have also been suggested in [34]. However, in this analysis process, some questionable assumptions were made. For instance, a Poisson-type arrival process for bursty machine-type communication traffic was assumed. These initial assumptions are critical as they influence the final results.

### 2.3. Architectural Improvements for RA

The unpredictable channel condition of mmWave networks results in a significant challenge of RA preamble detection in the physical layer. 5G and B5G services, including mMTC, evolved mobile broadband (eMBB), and URLLC, require efficiency and reliability of the initial attachment point. Because of severe congestion resulting from different mixed traffic sources originating from 5G networks, especially from mMTC, 3GPP introduced the concept of access class barring (ACB), where some users that would request access are barred from the service. The major concern regarding RA is the methods in which the access point dynamically selects RA control parameters, such as the number of preambles/number of consecutive preambles sent by users, ACB, and UBW, depending on the load size of the cell. The load is estimated based on past channel history, and the ACB parameter should be updated dynamically depending on the load size of each cell. The calculation of load estimation and the barring rate was proposed in [5,6].

To reduce RA delay, proposals have considered shortening the RA procedure from a four-way handshaking system to a two-way handshaking system, as illustrated in Figure 2. For example, the authors in [35] introduced the concept of a grant-free RA (one-shot) system, where data signals are sent along with the RA request. This is a viable solution where the data size is low for mMTC. A two-phase RA was proposed in [36] where users are first grouped according to the selected random preamble; for instance, users who select a certain preamble are in one group. No collision occurs across the group; thus, in the second phase, a dedicated channel is assigned for each group to request access. The concept allows the base station to decide the number of channels that should be assigned.

### 2.4. Perspective

Machine learning techniques do not assume predefined analytical formulation but rather help approximate complex stochastic functions based on stored information in the case of supervised learning, and trial-and-error setups in the case of reinforcement learning (RL), or a mixture of both. From the literature overview, we note that proposals have been developed on four-way handshaking for the RA procedure, and we believe that our machine learning procedure can be applied to any of the proposed architectural changes, depending on the considered use cases. To the best of our knowledge, it remains difficult to find machine learning techniques allowing users to predict congestion and thus select an appropriate access point without the help of a centralized controller. In light of this, the next section elaborates the problem formulation and our proposed system model.

## 3. Problem Formulation and System Model

**Figure 3:**
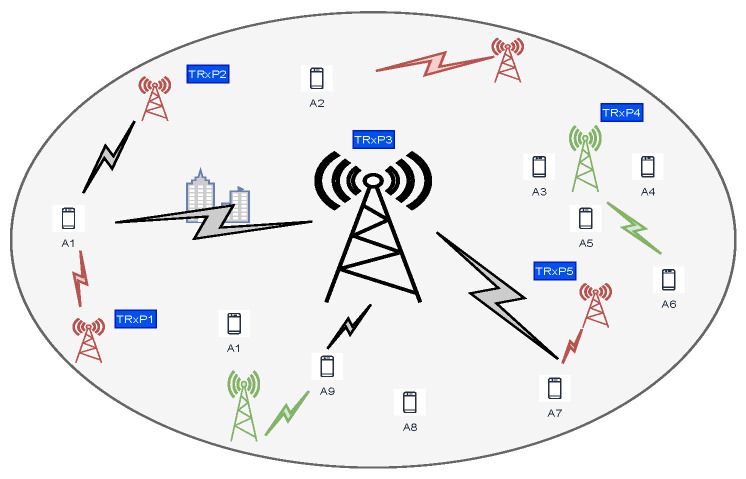
Users moving through the network have single or multiple selection options to perform a RA request depending on their position.

The system model is illustrated in Figure 3. The network model describes a scenario where multiple TRxPs $\left\{A_{1},A_{2},\ldots ,A_{N}\right\}$ are deployed to cover a large area. The TRxPs could be femto-cells, pico-cells, macro-cells, or gNodeBs. User devices $\left\{T_{1},T_{2},\ldots ,T_{K}\right\}$ requesting access should select one to proceed with the RA step. The users are moving within the network and therefore are constantly hopping among the TRxPs.

Users can receive a signal from one or more TRxP depending on their position. We do not assume a centralized controller managing the handover because it is unrealistic. In this case, users must identify TRxPs within the transmission range. Once the selection is performed, users participate in contention-based RA. The selection would be performed by using the deep reinforcement learning algorithm proposed in this paper. The TRxPs will rotate RAOs, which are the preambles periodically selected by the users. A four-way handshake or modified one-shot procedure can work with the considered system model.

### 3.1. Traffic Model

To evaluate the effectiveness of the proposed approach, we include a more congested scenario in the experiment using 3GPP’s synchronized traffic model 2 [37]. This refers to many machine-type devices accessing the network almost simultaneously. The traffic model from 3GPP allows us to experimentally model a congested network. The probability density function of the beta distribution is given as follows.

(1) $$g\left(t\right)=\frac{t^{\alpha -1}{\left(T-t\right)}^{\beta -1}}{T^{\left(\alpha +\beta -1\right)}B\left(\alpha ,\beta \right)},$$

(2) $$B\left(\alpha ,\beta \right)=\int_{0}^{1}t^{\alpha -1}{\left(1-t\right)}^{\beta -1}dt,$$

where *t* is the time of access opportunity at a given time, *T* is the duration of the activation of the devices, α and β are shape parameters of the beta distribution.

### 3.2. Combined Channel Model

Because of the tendency toward harsh channel statuses in mmWaves due to impairments such as obstructions, considerable research effort has been devoted to designing an accurate channel model for mmWave communications. The channel status is further degraded by the high mobility of users who frequently make handoffs between base stations. For this reason, a channel model accurately representing the network environment should be considered to capture and analyze the network scenario.

Based on real-world statistical measurements at frequencies of 28 and 73 GHz in New York City, the authors in [38] presented a channel model for dense urban deployment. The path loss measurements adopted in this paper are as follows.

(3) $$\begin{array}{c} {PL}_{LOS}\left(dB\right)=\alpha +\beta {10\log}_{a}\left(d\right)+\xi_{c},\\ \xi_{c}\overset{}{∼}N\left(0,\theta \right), \end{array}$$

where *d* is the distance, α and β are the least-square fits of floating intercept and slope over a covered distance, respectively. $\xi_{c}$ is the log–normal shadowing. The shadowing variance is given by θ.

### 3.3. Problem Formulation

Any of the models proposed for RA enhancement can be assumed. In a four-way handshake RA, users send preambles at the first step. In three subsequent steps, TRxPs and users exchange control information leveraging resource reservation and collision resolution. For a one-shot-based and two-way RA, data can be sent along with the RA request in two steps. For these models, prior works required separate mathematical analyses to provide an analytical expression of the RA delay. However, in this work, we do not assume a predefined expression, and the analysis presented can be applied to any of these models.

As shown in the system model in Figure 3, when a user moves through a TRxP’s coverage area, it receives a signal from a single TRxP or some subset depending on the position. It also contends with other users to seize an RA preamble opportunity. We consider a selection problem where the goal is to connect with a less congested TRxP; therefore, to reduce the amount of time the users spend to perform the RA. We also assessed other RA performance metrics.

The problem statement is that we need to select an optimal policy that maximizes the probability of selecting a single best performing TRxP in a given spatial and temporal situation out of the *K* TRxPs available for the users to choose from. In such a situation where the user receives multiple signals from the available access points, the user can estimate the congestion level indirectly by ranking the TRxPs according to a delay variable representing an expectation of RA completion time for each. Considering this, users can select the optimal access point.

We consider that as users move throughout a network of dense TRxPs, $\left\{A_{1},A_{2},\ldots ,A_{n}\right\}$, where at any given RAO time, $t_{i}$, and position in the network, a user has some subset of TRxP choices of which to consider requesting RA, one TRxP is selected. Weights for each TRxP are assigned by the users for the purpose of the selection; thus, the higher the weight of a TRxP, the higher the chance of it being selected. Therefore, the probability that h-TRxP is selected depends on its weight and is given by:

(4) $$\frac{w_{i}}{\sum_{\forall i}w_{i}}.$$

We consider four strategies to obtain a policy that maximizes the selection strategy, which is equivalent to maximizing the probability of selecting a better-performing TRxP. Four strategies for selection are considered, including (1) A strategy that randomly selects one of the available TRxPs, where each has an equal probability of selection, (2) a strategy that considers a channel quality metric based on reference signal received power (RSRP) measurements of users at the current time slot, ranking the TRxPs accordingly, (3) a weight ranking strategy, where weights are assigned based on the previous time slot experiences of a user, and (4) a strategy that considers any combination of the above strategies. A subset of the *n* TRxPs are available for a user to select at a given point, and the following weights are assigned to those members: $\left\{w_{1},w_{2},\ldots ,w_{n}\right\}$.

If we consider the users’ experience to rank TRxPs, a weight, $w_{i}$, of any TRxP can be calculated from the equation given below.

(5) $$w_{i}=d_{t-1,a_{i}}=\left\{\begin{array}{cc} T_{4a_{i}}-T_{1a_{i}}, & \mathrm{in}\;\mathrm{four}\;\mathrm{way}\;\mathrm{RA},\\ T_{2a_{i}}-T_{1a_{i}}, & \mathrm{in}\;\mathrm{two}\;\mathrm{way}\;\mathrm{RA}, \end{array}\right.$$

where $d_{t-1,a_{i}}$ is the user delay experience at a given RAO slot with TRxP *i*, $T_{4a_{i}}$ is the time the user completed step four, $T_{2a_{i}}$ is the time the user completed step two, and $T_{1a_{i}}$ is the time the user initiated the RA procedure. Let $D_{t}$ represent a function encoding all the weights from all available TrXPs as follows.

(6) $$D_{t}=x_{1}d_{t-1,a_{1}}+x_{2}d_{t-1,a_{2}}+\cdots +x_{n}d_{t-1,a_{n}}.$$

The variables $x_{1},x_{2},\ldots ,x_{n}$ are natural numbers such that:

(7) $$\begin{array}{c} x_{1}+x_{2}+\cdots +x_{n}=1,\\ x_{1}x_{2}\ldots x_{n}=0. \end{array}$$

The optimal TRxP selection strategy is given by the following optimization equation.

(8) $$\begin{array}{cc} \arg\min_{a\in A}D_{t} & \\ \mathrm{subject}\;\mathrm{to}\; & \left(1\right)P_{a_{1}}\geq P_{t_{1}},\\ & \left(2\right)P_{a_{2}}\geq P_{t_{2}},\\ & \;\vdots \\ & \left(3\right)P_{a_{n}}\geq P_{t_{n}},\\ & \left(4\right)R_{\alpha }\geq P_{\alpha },\\ & (5)P(W\geq t)\leq \delta . \end{array}$$

The parameters in the constraints are defined as follows. Constraints (1)–(3) are related to the power strength received from the available TrXPs. $P_{a_{i}}$ is the power received from TRxP $a_{i}$, which should be at least equal to the threshold value $P_{t}$. This constraint requires that users receive an acceptable power level from the TRxP to consider it for selection and later perform RA through it. In constraint (4), users draw a random number, $R_{\alpha }$, that should be greater than the system parameter, $P_{\alpha }$, broadcast by the TRxP, enforcing a prohibition on excessively massive connection scenarios for accepted RA requests. Before considering the current TRxP for selection, users must first make sure they pass this requirement.

Constraint (5) describes the delay-budget of the user. Weight (delay) estimation is another task in solving the equation given in (8). An exact analytical expression representing the weights first depends on the RACH model, and second, considers strong assumptions from queuing theory. In this study, however, we employ learning techniques that enable the proposed method to predict (estimate) the weights, which also can be advantageous in that they do not assume a predefined RACH model. A deep RL formulation for the delay is provided in the next section.

## 4. RL Based Selection of TRxPs for RA

In this section we present a comprehensive analysis in which we sequentially address the initial steps of RA, along with some of the proposals suggested to optimize RA channel performance.

### 4.1. TRxPs Search and Selection

(1) The cell search procedure is initiated when a user has a buffer to send/receive data to/from the TRxPs. Cell search and selection criteria depend on the users’ RSRP and reference signal received quality (RSRQ) measurements. Based on these measurements, users can select from the available TRxPs and monitor system parameters through the system information block (SIB2) signal. A user can attach to a selected TRxP given that its current RSRP measurement is higher than a threshold value provided according to the following equation.

(9) $$\mathrm{RSRP}>{\mathrm{RSRP}}_{\min}.$$

### 4.2. System Parameters through SIB2

(2) UBW and ACB are random variables that are initially broadcast by the TRxPs and later selected by the users to reduce the occurrence of collision and congestion, respectively. UBW is the original standard of RA. ACB is a recently adopted mechanism to control congestion in massive RA scenarios. They become important inference variables for learning because it is shown that proper selection of these parameters allows reduction of collision and congestion, and therefore has a direct impact on estimating the RA delay. Once users select the proper system parameters, the available resources for RA are made known to them according to the configuration from the TRxPs.

### 4.3. Proposed RL Based Selection for RA

**Figure 4:**
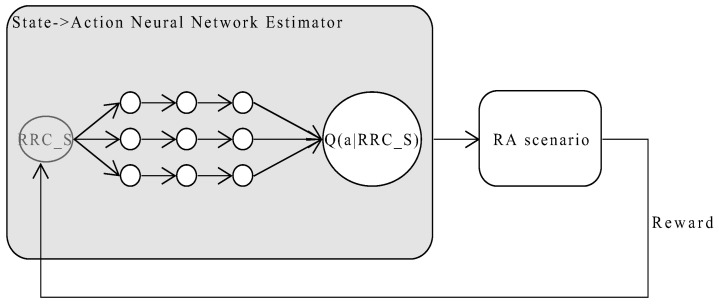
The Deep *Q*-Network (DQN)-based agent interacts with the random access network environment to receive rewards.

In the RL algorithm, users learn the selection policy through interaction with the RA environment, as depicted in Figure 4. When a user needs to establish/re-establish a network connection with a TRxP or the buffer is ready, the learning algorithm selects a TRxP before proceeding to the RA procedure. This is done before exchanging message 1 in the RA protocol procedure, illustrated in Figure 2. The user observes the state, takes an action, receives a reward, and observes the next state along with their current connection status. The reward is a factor of the RA’s key performance indicators (KPIs), explained in detail in Section 4.4.2. In general, it combines values from both the state and action sets with KPIs. This can be represented by using a Markov decision process, as in a simple tuple $\left(S,A,r,S^{{}^{\prime }}\right)$, where *S* is the state space, *A* is the action space, *r* is the reward, and $S^{{}^{\prime }}$ is the new state. An agent is initially at a given state. It takes action and receives a reward. After that, it transits to the next state. Transition probabilities are not predetermined.

The goal of such users is to maximize their expected long-term reward value. The KPI values can be accumulated to measure whether users are maximizing their reward. Starting from a certain position, where the network starts, the expected long-term reward can be formulated as in the following equation.

(10) $$E\left[r\right]=\sum_{i=0}^{\infty }\left(\gamma_{i}S_{i}|S=S_{0}\right),$$

where $S_{0}$ is the initial state, $S_{i}$ is the ith state, and γ<1.

The action value function estimation is based on a neural network (thus deep RL) and allows the selection of the best action, which refers to the selection of the better performing access point. In the case of *Q*-based tabular learning, we can store the values in a *Q*-table initialized to null or infinity values. As the agent traverses through the network and gains information, the table is updated progressively. For deep RL, the algorithm itself approximates this action value function.

We approach the problem by solving the deep RL task for two main reasons. First, the difficulty of the *Q*-based tabular algorithm in estimating new unseen positions, and second, the complexity of maintaining the table with increasing network size. Therefore, instead of using empty tables for the value-function approximation or using only *Q* tables, our neural network approach estimates the state-action-value function without the need for *Q* tables.

The agent performs neural-network-based RL. It selects the optimal actions; that is, a user must be attached to a nearby cell based on the stored experience and current state. The agent also performs exploration with a certain probability and receives rewards in terms of the delay experienced by the user during the RA request. The reward is then recorded to update past experiences. In this manner, the agent makes predictions as well as learns from past experiences.

Finally, we can find the optimal policy by evaluating the optimal *Q* value, given in Equation (11) below. Q(S,a) measures the importance of taking action *a* in state *S*. Out of the many actions listed, the optimal policy selects the optimal *Q* value in the following manner. During exploitation, it selects an action with a probability of (1−ϵ), and during exploration, with a probability of ϵ. Exploration is performed at the beginning of the training. As the agent advances through training, ϵ decays with a decaying factor so that exploitation of the experiences observed so far begins to be utilized. To summarize, Q(S,a) is updated as follows.

(11) $$Q\left(S,a\right)=\alpha \left(r+\gamma \arg\;\min Q\left(S^{{}^{\prime }},a\right)\right)+\left(1-\alpha \right)Q\left(S,a\right).$$

As we are considering the RA delay as a factor for determining the reward values, the policy optimization can be safely written as a minimization function given as

(12) $$\pi =\arg\min_{a\in A}Q^{*}\left(S,a\right).$$

In deep RL, we approximate the above *Q* function by minimizing the error between the estimator and the *Q* function given below.

(13) $$Error={\left(Q\left(s,a,\theta \right)-Q\left(s,a\right)\right)}^{2},$$

where θ is the weights from the neural network.

### 4.4. Design

The architecture of the proposed neural network-based RL model is depicted in Figure 4. The agents running the deep RL algorithm interact with the RA environment by taking action from the action set. The RA environment in turn responds by returning a reward, which is a factor of the RA KPIs. The input to the network is the current state of the user from RRC_IDLE, RRC_INACTIVE or RRC_CONNECTED. To rearrange the dataset, we associate simple numerical values to the various RA states a node can occupy. We seek to obtain estimates of state-value action as an output, and the learning algorithm uses these metrics to select the best performing access point for an RA request.

As with a typical RL design, we list the entities as follows.

#### 4.4.1. States

A single state in our design is further composed of three entities. These are the initial criteria, system parameter criteria, and set of TRxPs. We further elaborate on the entities as follows:

- Initial criteria: In the RA selection problem, user nodes are allowed to participate in the RA selection problem if they are in the RRC_IDLE RA mode and have a full buffer to send data in the uplink, or a request from a TRxP to receive data in the downlink. Mathematically, we use an indicator variable as follows.
  (14) $$I=\left\{\begin{array}{cc} 1 & if\;\mathrm{user}\;\mathrm{has}\;\mathrm{full}\;\mathrm{buffer},\\ 0 & else. \end{array}\right.$$
- System parameters criteria: Once users pass the above criteria, the next step is to pass the ACB system parameter criteria that they receive from the serving cell. The ACB flag indicates whether users are barred from performing RA in case of insufficient resources. Initially, this parameter is represented as a real number. We use another indicator variable to check whether a user has passed this parameter as follows.
  (15) $$A=\left\{\begin{array}{cc} 1 & if\;0<a<t<1,\\ 0 & else, \end{array}\right.$$
  where *a* is the ACB factor the user randomly selects and sends to the serving cell, and *t* is a threshold factor for admission control by the cell. Finally, the user is allowed to perform RA if the above two criteria are successfully met, which is mathematically presented as
  (16) $$E=\left\{\begin{array}{cc} 1 & if\;I==1\&A==1,\\ 0 & else. \end{array}\right.\;$$
- Set of TRxPs: We encode the set consisting of the TRxPs from which user nodes can obtain a pilot signal and measure the RSRP representing potential candidates for RA channel selection. This is given as follows.
  (17) $$A=<A_{1},A_{2},\ldots ,A_{n}>,$$
  where
  (18) $$A_{i}=\left\{\begin{array}{cc} 1 & if\;T_{i}\;\mathrm{is}\;\mathrm{within}\;\mathrm{reach},\\ 0 & else. \end{array}\right.\;$$

Finally, the state is represented by the vector as:

(19) S=[E,A].

#### 4.4.2. Reward Function

The reward obtained from the RA environment can be thought of as having an inverse relationship with the RA delay in successfully completing a procedure. A higher reward value means that the user completed the RA procedure with less delay and even within a shorter time before the delay budget expires. The reward value also encodes the delay budget given below. For instance, if a user cannot complete a random access within the delay limit, the reward value approaches zero. Otherwise, the reward is calculated as the inverse of the completion time. Mathematically, it is given by the following equation:

(20) $$r=1/D,\left\{\begin{array}{cc} D=D_{i} & \mathrm{if}\;P(W\geq t)\leq \delta ,\\ D\to \infty & \mathrm{else}, \end{array}\right.$$

where $D_{i}$ is the delay user experienced from TRxP *i*. *W* is the waiting time of the user, and δ, a delay threshold.

#### 4.4.3. Using Replay Buffer for Stability

We use experience replay for efficiently running the stochastic gradient descent algorithm updating weights in the neural network. This approximates the *Q* function given in Equation (11) by minimizing the loss function given in Equation (21) below. In addition, the channel condition of the network is dynamic. The state-action values used for initial training may change over time, and therefore, it is more appropriate to sample experiences. Therefore, we store some state-action-value pairs annexed by time slots as given in Equations (22)–(24) in which the agent samples from at a later point in time to decide on the actions to perform.

(21) $$Loss=\sum_{i=1}^{n}{\left(Q\left(s,a,\theta \right)-Q\left(s,a\right)\right)}^{2},$$

where θ is the weights from the neural network.

(22) $$\begin{array}{c} s_{1}=<E,T,T_{1},D_{1},RRC\_C,t_{1}>, \end{array}$$

(23) $$\begin{array}{c} s_{2}=<E,T,T_{2},D_{2},RRC\_C,t_{2}>, \end{array}$$

                                               ⋮

(24) $$\begin{array}{c} sn=<E,T,T_{n},D_{n},RRC\_C,t_{n}>. \end{array}$$

Because the nature of the network is dynamic, it is not sufficient if only the last experience is considered for making predictions; rather, a random batch of experiences is selected from storage and helps the machine agent learn from long-term experiences.

### 4.5. Algorithms

In this section, we present the pseudocode for the four algorithms proposed and analyze their performance through a comparison. Algorithms 1 and 2 describe our proposals in this work. Algorithm 3 is a *Q*-based algorithm [39], and Algorithm 4 is a random TRxP selector. The baseline algorithm is the original 3GPP selection algorithm, which selects a TRxP based solely on the RSRP measurement, i.e., a user ranks and assigns a numerical weight to each TRxP that it receives a pilot signal from. Finally, the TRxP that returned the highest rank was selected for the RA procedure.
**Algorithm 1** DQN-based Intelligent TRxPs Selector Algorithm: Training.1: Initialize discount rate (γ∈(0,1)), ϵ-greedy (ϵ∈(0,1)) rate value, and the range of an episode2: Start RA in a mobile network3: **while** (Episode is not finished) **do**4:   **while** (Not every position is explored or step is reached) **do**5:     Get the visible TRxPs at the current position according to Equation (9)6:     Select a TRxP based on the ϵ-greedy policy; predict a TRxP returning the minimum RA delay from the deep neural network or select a random TRxP.7:     Receive the reward according to Equation (20)8:     Remember (CurrentPosition, selectedTRxP, reward, nextSelectedPosition, trainingEndMarker)9:     Replay by sampling the experiences obtained from the above steps from 5 to 810:   Train by updating the weights of the DQN11: **end while**12:**end while**

**Algorithm 2** DQN-based Intelligent TRxPs Selector Algorithm: Online.
1: **while** (getRRCState() == RRC_IDLE && UE has buffer) **do**2:  Get the visible TRxPs at the current position according to Equation (9) and calculate the state3:  Feed the state input to Algorithm 14:  Get the *Q* values of every TRxPs and select the maximum5:  Perform RA with selected TRxP and receive reward6:  Store reward7: **end while**8: **while** (getRRCState() == RRC_IDLE) **do**9:  Run Algorithm 110:
**end while**



The online algorithm given in Algorithm 3 is based on a tabular *Q* value function. It updates each access point (TRxP here) according to Equation (25) given below. In Algorithm 1, the agent is trained for a number of episodes. In each step of a single episode, it aims to explore as many user positions as possible to gain an understanding of the network environment. Each selection decision moves the state of the agent from RRC_IDLE mode to RRC_Connected and then back to the former, to explore more positions.

The Deep *Q*-Network (DQN) algorithm has two neural networks in the implementation. One estimates the *Q* value and transfers the learned weights to the other neural network. Finally, a mini-batch of experiences is sampled, and the neural network is trained on the updated information. The online algorithm given in Algorithm 2 makes use of the output obtained from Algorithm 1. Algorithm 2 executes when the user needs to perform the RA (i.e., it has a full buffer and passed system parameter criterion). To further minimize delay, additional training on the updated environment experiences is run only when the user is in RRC_IDLE mode. Here, Q(s,a) is updated as follows.

(25) Q(S,a)=αr+(1−α)Q(S,a).

**Algorithm 3** *Q*-Based Intelligent TRxPs Selector Algorithm: Online.
1: Initialize discount rate (γ∈(0,1)), ϵ-greedy (ϵ∈(0,1)) rate value, and the range of an episode2: Start the RA network3: **while** (Episode is not finished) **do**4:  **while** (Not every position is explored or steps is reached) **do**5:    Get the visible TRxPs at the current position according to the equation given in (9)6:    **if** (empty *Q* table) **then**7:      Select a random TRxP8:      Perform RA with selected TRxP, receive reward and update the *Q* value of that TRxP9:    **else**10:     Select a TRxP based on an ϵ-greedy policy; get the TRxP that returned highest *Q* value or select a random TRxP.11:     Receive the reward according to Equation (20)12:     Update *Q* value and get the next state13:   **end if**14:  **end while**15:
**end while**



**Algorithm 4** Random TRxPs Selector Algorithm: Online.
1:**while** (getRRCState() == RRC_IDLE && UE has buffer) **do**2:  Get the visible TRxPs at the current position according to Equation (9) and calculate the state3:  Select a random TRxP4:  Perform RA with selected TRxP and receive reward5:
**end while**



## 5. Evaluation

For the purpose of evaluating the proposed algorithm and analyzing the performance gains compared to other algorithms, we conduct experiments with a well-known simulator, ns-3 [40]. We also used Python to implement the proposed RL algorithm. The main simulation and analytical parameters are explained in the following subsections. We consider two main criteria: (1) learning performance and (2) the algorithms’ relative performance compared to other previous proposals, including *Q*-based algorithms, RSRP-based selection algorithms, and random selection algorithms.

### 5.1. Experimental Setup

For simulation, we used ns-3. We simulated a random access network environment, and Table 1 presents some of the main parameters used. We consider the contention-based RA, where the TRxPs do not pre-allocate resources for users, users compete to seize an RA opportunity, and collisions occur in doing so. We also assume that users frequently trigger the RA and re-establish connections as in highly mobile environments. The mobility pattern follows a random uniform distribution. Each RA slot includes six physical resource blocks (PRBs), totaling 1.08 MHz. The number of available RA preambles in an RAO is 64. Users send a maximum of 50 preambles before they assume that the network is unavailable and then withdraw. The average tolerable delay is 100 slots, and the TRxPs can use any scheduler. In our case, we adopt the proportional fair scheduler. The main parameters used for the deep RL algorithm are summarized in Table 2. We use keras over Tensor-flow for the implementation. There are four connected layers. Each hidden layer has 48 neurons. Neurons are activated according to a Relu activation function.

### 5.2. Performance Metric Measures

The performance was obtained from the perspective of RA delay, successful access probability (SP), and waiting time distribution. Delay measures the time difference between when users seize an RAO, and when the decision notification by the access point arrives. More technically, in a four-way RA, it is the time difference between messages 1 and 4; and in a two-way RA, it is the difference between messages 1 and 2 of the RA procedure. SP measures the chance of successful transmission of a user given that many users are competing to seize an RA request preamble (opportunity). The number of RA preambles sent gives the number of times the user has been keeping retrials before access is granted.

A normalized cumulative reward function for a number of episodes can be formulated as follows:

(26) $$R=1/\sum_{i=1}^{\left|E\right|}1/D_{i},$$

where |E| represents the magnitude of the episodes per iteration.

### 5.3. Learning Performance

**Figure 5:**
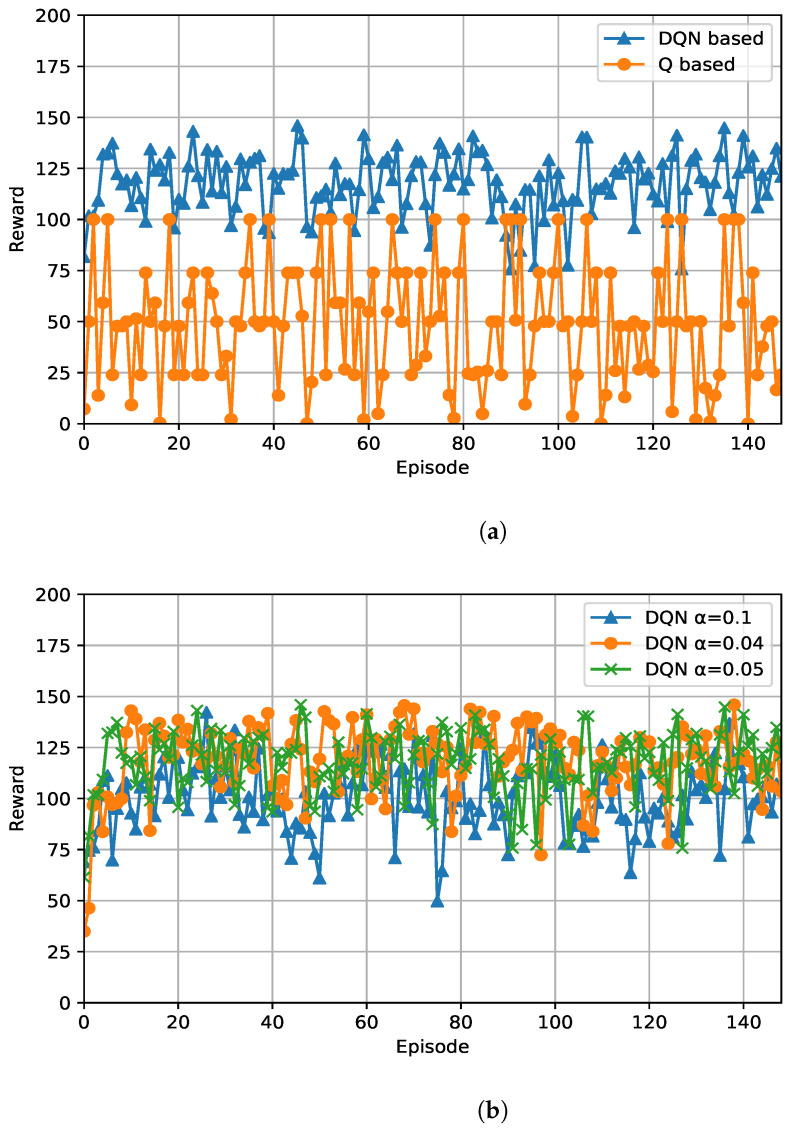
Convergence plot of *Q*-based and DQN-based algorithms: stability of reward values despite a random access network environment. Reward values are computed for every other episode. (**a**) Comparison between RL approaches for reward values in random access network environment; (**b**) reward values for DQN-based algorithm: different learning rates.

The goal of each learning user is to choose the best serving TRxP among the many TRxPs from which it gets an RAO advertisement. Selecting the best serving TRxP allows the user to perform the RA procedure quickly, and transition into the phase of radio resource scheduling. Intuitively, this will be the least congested TRxP. Figure 5a shows the performance of the learning algorithm. It is observed that the performance increases and the reward becomes stable.

We averaged the reward values for every other episode. The algorithm quickly reaches its peak reward value around the 20th episode. Subsequently, it tries to maintain this value despite the randomness of the environment. The algorithm’s performance does not dip any lower than the initial reward value afterwards. The effect of the learning rate on the DQN algorithm is shown in Figure 5b. A lower learning rate eventually allows for better performance improvement despite the initial penalty. Initially, it is desirable that the algorithm relies on learning instead of exploiting its inadequate experience, and thus it shows a low performance. After a while, the learning rate should be smaller, and the users should be able to exploit their experience for better performance.

### 5.4. Impact of Proposed Algorithm on RA KPIs

In this subsection, the impact of the DQN on the RA delay, SP, and waiting time distribution is analyzed.

#### 5.4.1. Reliability

**Figure 6:**
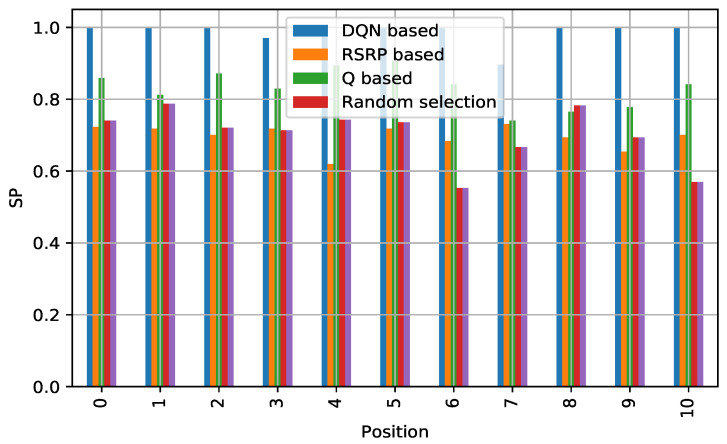
Comparison of successful access probability: DQN-based vs. others in random access network environment.

Figure 6 depicts the SP of the proposed algorithm against the others. To describe the test results for the learning curve as well as the performance on improving SP, the figure shows different positions where the metric is calculated. The position can be random; however, we test the initial position as well to test how the SP improves, which helps to also visualize how the algorithm is learning. We observe that, in the initial positions, because the network is starting, and previously-stored information is not available, the deep RL algorithm proceeds to exploit as expected, and in the remaining positions, we observe that the deep RL method outperforms the other algorithms in terms of reliability. The increase in SP is attributed to the algorithm’s efficient way of selecting a less congested TRxP by exploiting its own stored knowledge.

#### 5.4.2. RA Delay

**Figure 7:**
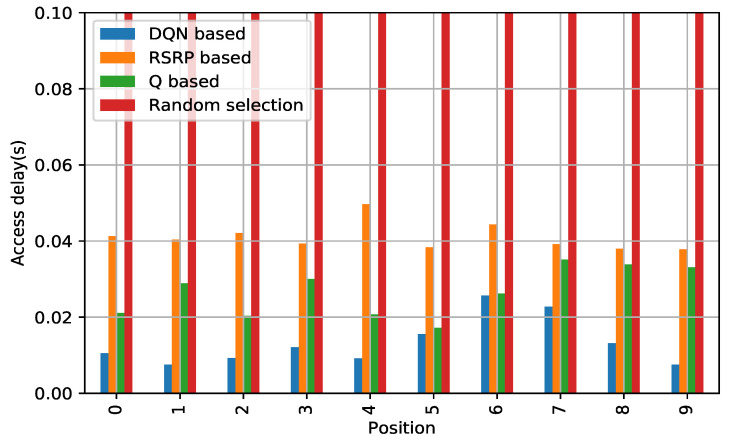
Comparison of access delay: DQN based vs. others in random access network environment.

We also test the RA delay, measured from different positions. We find that the DQN-based algorithm has superior performance compared to algorithms for different positions, as shown in Figure 7. The same reasoning follows from that described for the reliability metrics. Generally, some performance degradation is observed for DQN at some locations. This is because of the dynamic environment, as expected. Although the *Q*-based algorithm shows a reasonable performance, DQN shows the most promising performance on average. Particularly, in comparison with the current 3GPP’s methodology of selection, The DQN-based algorithm reduced the RA delay by 58.89%.

#### 5.4.3. Waiting Time Distribution

**Figure 8:**
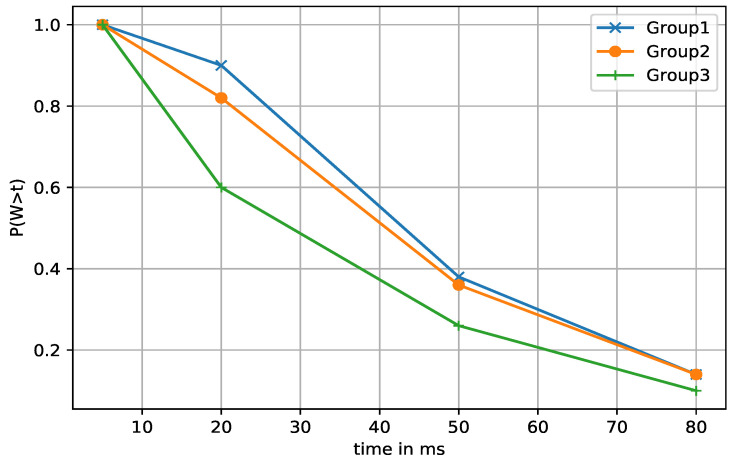
DQN’s waiting time distribution measured at different groups of episodes in random access network environment.

**Figure 9:**
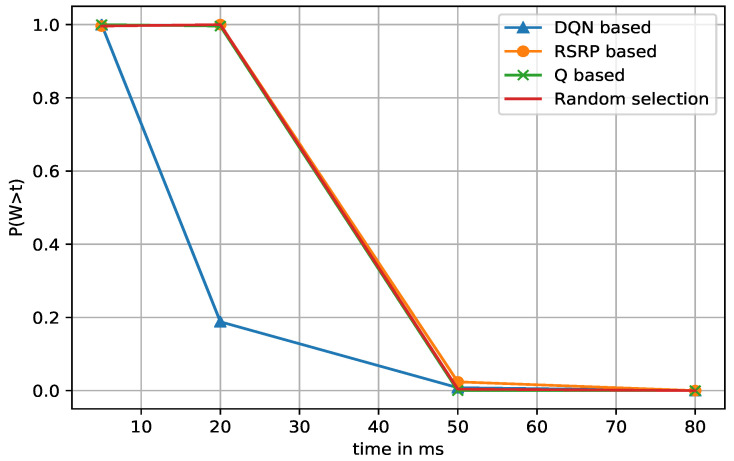
Comparison of waiting time distribution: DQN-based vs. others in random access network environment.

The dropped packet rate can be explained in terms of the waiting time distribution. It could be defined as the probability that the waiting time of a request does not exceed the delay budget of the user. We measured the waiting time distribution for different delay budgets of packets at different time points. In Figure 8, we grouped the episodes into 50 units. Group 3 requires more training time. The DQN allowed the waiting time to decrease sharply, which also means fewer drop rates. The sharp decrease is more pronounced for the latter groups. In addition, Figure 9 illustrates the comparison between DQN and the other algorithms on the waiting time distribution. DQN again shows the best results.

#### 5.4.4. Algorithm Overhead Analysis

Generally, the potential drawback of learning algorithms is the resulting overhead from implementation when the algorithm makes an inference. We discuss the overhead of the proposed DQN algorithm in terms of the storage(space) and running time. Our deep reinforcement learning algorithm, as applied to the aforementioned environment, has a possible advantage in terms of memory overhead over other algorithms, such as Q learning. This is because it does not store the increasing number of state and action pairs for training. It rather approximates that function, and we use replay memory for storing experiences as Algorithm 1 illustrates. Then, a min-batch size of the memory is used for experience replay. Therefore, space complexity is minimal.

In spite of that, we need to store samples of experiences. This depends on the selected batch memory size, which can be fixed (the size doesn’t grow) because of being overwritten frequently and storing the most recent batch of experiences. Allowing more size helps the agent to sample from more experiences and cancels correlation, which is helpful for the stability of the algorithm. Therefore, the memory overhead is upper bounded by the replay memory size *M*. Let *M* represent the memory replay size, and the number of bits required to store our current state, next state, the action taken is *B* bits. Therefore, the RAM memory overhead $R_{m}$ of the devices is given by the following equation.

(27) $$R_{m}=M*B.$$

For inference, Each layer has a matrix of weights. The size reserved for one of the matrices can be used for the next multiplication operation performed at the next layer. Therefore, space is bounded by the size of the biggest weight matrix. It is given by:

O(nk),

where *n* and *k* are the dimensions of the rows and columns of the matrix respectively. Time overhead in forward-propagation constitutes matrix multiplication operations. Suppose *n* is the number of layers. Matrix multiplication operation performed at each layer is in the $O(n^{3})$ time complexity. The overall time overhead is given by:

(28) $$\left(n-1\right)layers*n^{3}=O\left(n^{4}\right).$$

## 6. Conclusions

For random access in 5G and B5G networks, users have the opportunity to receive pilot signals from a number of neighboring TRxPs. This paper proposes and tests the feasibility of recent machine learning approaches, in particular RL, to solve a random access network control problem. If we allow users to have stored knowledge (through training) in terms of the service points that show better performance, they can opportunistically choose the optimal access point, which helps optimize their expected RA performance. We conducted an experiment using ns-3 to prove the efficacy of our proposed RL method in a dynamic network scenario with channel conditions varying over time.

There are significant implications of applying the reinforcement learning method for wireless networking. For example, it allows us to estimate the random access delay that occurs at a given TRxP without requiring an exact model of the random access environment since users learn about the environment through their own experience. Another benefit for future networks is that users can intelligently determine the optimal attachment points in such a way that their QoS can be met without requiring a central agent to calculate their expectations and provide resources accordingly.

The next step after random access is a data exchange. Such a learning algorithm, for example, reduces the congestion that can occur at the would-be overloaded access points. Hence, access request load is shared among the access points. Learning algorithms will determine the load-sharing strategy without requiring a model. In this way, efficient resource distribution and allocation are realized throughout the whole coverage area.

The potential drawback of learning algorithms is the overhead. Space complexity is minimal. However, despite a slight training time penalty, the deep RL algorithm increases the overall long-term reward values for the users. We further analyze such learning algorithms to achieve better convergence and performance results in fewer iterations, and also reduce the associated computational overhead, which is left as an avenue for our future work.

## Figures and Tables

**Table 1:** Simulation parameters.

Parameter	Value
Maximum number of retrials	50
Number of available preambles	64
Number of access points	5
Tolerable delay	100 slots
Scheduler	Proportional Fair
Channel frequency	28 GHz
Total number of users	900
Number of TRxPs	5
Mobility pattern	ConstantVelocityMobilityModel
Position allocator	Random uniform distribution allocator
Scheduler	Proportional Fair

**Table 2:** The parameters used for DQN.

Parameter (Description)	Value
*m* (Replay memory size)	500,000
*M* (Mini-batch size)	32
γ (Discount factor)	0.95
ϵ	(0.01, 1.0)
$\epsilon_{decay}$	0.0001
α (Learning rate)	0.0001
τ (Copy rate)	0.05
Optimizer	Adam
Activation	0.05
Episodes	1000
Steps	500
Connected layers	4

## Data Availability

Not applicable.

## References

[1] (2011). 3rd Generation Partnership Project; Technical Specification Group Radio Access Network; Evolved Universal Terrestrial Radio Access (E-UTRA); Radio Resource Control (RRC); Protocol Specification. https://www.3gpp.org/ftp/Specs/archive/36_series/36.331.

[2] (2011). 3rd Generation Partnership Project; Radio Resource Control (RRC); Protocol Specification. https://www.3gpp.org/ftp/Specs/archive/38_series/38.331.

[3] (2011). 3rd Generation Partnership Project; Technical Specification Group Radio Access Network; Evolved Universal Terrestrial Radio Access (E-UTRA) User Equipment (UE) Procedures in idle Mode. https://www.3gpp.org/ftp/Specs/archive/36_series/36.304.

[4] (2011). 3rd Generation Partnership Project; Technical Specification Group Core Network; NAS Functions related to Mobile Station (MS) in idle Mode. https://www.3gpp.org/ftp/Specs/archive/23_series/23.122.

[5] Wan C., Sun J. Access Class Barring Parameter Adaptation Based on Load Estimation Model for mMTC in LTE-A. Proceedings of the 2019 International Conference on Communications, Information System and Computer Engineering (CISCE).

[6] Tello-Oquendo L., Vidal J.R., Pla V., Guijarro L. Dynamic access class barring parameter tuning in LTE-A networks with massive M2M traffic. Proceedings of the 2018 17th Annual Mediterranean Ad Hoc Networking Workshop (Med-Hoc-Net).

[7] Sutton R.S., Barto A.G. (2018). Reinforcement Learning: An Introduction.

[8] Watkins C.J., Dayan P. (1992). Q-learning. Mach. Learn..

[9] Mnih V., Kavukcuoglu K., Silver D., Rusu A.A., Veness J., Bellemare M.G., Graves A., Riedmiller M., Fidjeland A.K., Ostrovski G. (2015). Human-level control through deep reinforcement learning. Nature.

[10] Kaelbling L.P., Littman M.L., Moore A.W. (1996). Reinforcement learning: A survey. J. Artif. Intell. Res..

[11] Puterman M.L. (2014). Markov Decision Processes: Discrete Stochastic Dynamic Programming.

[12] Russell S., Norvig P. Artificial Intelligence: A Modern Approach. https://storage.googleapis.com/pub-tools-public-publication-data/pdf/27702.pdf.

[13] Mnih V., Kavukcuoglu K., Silver D., Graves A., Antonoglou I., Wierstra D., Riedmiller M. (2013). Playing atari with deep reinforcement learning. arXiv.

[14] Lillicrap T.P., Hunt J.J., Pritzel A., Heess N., Erez T., Tassa Y., Silver D., Wierstra D. (2015). Continuous control with deep reinforcement learning. arXiv.

[15] Van Hasselt H., Guez A., Silver D. Deep reinforcement learning with double q-learning. Proceedings of the AAAI Conference on Artificial Intelligence 2016.

[16] Wang C.X., Di Renzo M., Stanczak S., Wang S., Larsson E.G. (2020). Artificial intelligence enabled wireless networking for 5G and beyond: Recent advances and future challenges. IEEE Wirel. Commun..

[17] Kulin M., Kazaz T., De Poorter E., Moerman I. (2021). A survey on machine learning-based performance improvement of wireless networks: PHY, MAC and network layer. Electronics.

[18] Asadi A., Müller S., Sim G.H., Klein A., Hollick M. FML: Fast machine learning for 5G mmWave vehicular communications. Proceedings of the IEEE INFOCOM 2018—IEEE Conference on Computer Communications.

[19] Cao X., Ma R., Liu L., Shi H., Cheng Y., Sun C. (2018). A Machine Learning-Based Algorithm for Joint Scheduling and Power Control in Wireless Networks. IEEE Internet Things J..

[20] Zhang C., Patras P., Haddadi H. (2019). Deep learning in mobile and wireless networking: A survey. IEEE Commun. Surv. Tutor..

[21] Khan M.A., Kim J. (2020). Toward Developing Efficient Conv-AE-Based Intrusion Detection System Using Heterogeneous Dataset. Electronics.

[22] Luong N.C., Hoang D.T., Gong S., Niyato D., Wang P., Liang Y.C., Kim D.I. (2019). Applications of deep reinforcement learning in communications and networking: A survey. IEEE Commun. Surv. Tutor..

[23] Xiong Z., Zhang Y., Niyato D., Deng R., Wang P., Wang L.C. (2019). Deep reinforcement learning for mobile 5G and beyond: Fundamentals, applications, and challenges. IEEE Veh. Technol. Mag..

[24] Wang J., Zhao L., Liu J., Kato N. (2019). Smart resource allocation for mobile edge computing: A deep reinforcement learning approach. IEEE Trans. Emerg. Top. Comput..

[25] Orsino A., Galinina O., Andreev S., Yilmaz O.N., Tirronen T., Torsner J., Koucheryavy Y. Improving initial access reliability of 5G mmWave cellular in massive V2X communications scenarios. Proceedings of the 2018 IEEE International Conference on Communications (ICC).

[26] Galinina O., Turlikov A., Andreev S., Koucheryavy Y. Multi-channel random access with replications. Proceedings of the 2017 IEEE International Symposium on Information Theory (ISIT).

[27] Grassi A., Piro G., Boggia G. (2018). A look at random access for machine-type communications in 5th generation cellular networks. Internet Technol. Lett..

[28] Alavikia Z., Ghasemi A. (2019). Pool resource management based on early collision detection in random access of massive MTC over LTE. Ad Hoc Netw..

[29] Sinitsyn I.E., Zaripova E.R., Gaidamaka Y.V., Shorgin V.S. (2018). Success Access Probability Analysis Using Virtual Preambles Via Random Access Channel. CEUR Workshop Proceedings.

[30] Yuan J., Huang A., Shan H., Quek T.Q., Yu G. (2018). Design and Analysis of Random Access for Standalone LTE-U Systems. IEEE Trans. Veh. Technol..

[31] Agiwal M., Qu M., Jin H. Abstraction of Random Access Procedure for Bursty MTC Traffic in 5G Networks. Proceedings of the 2018 24th Asia-Pacific Conference on Communications (APCC).

[32] Park S., Lee S., Choi W. Markov Chain Analysis for Compressed Sensing based Random Access in Cellular Systems. Proceedings of the 2019 International Conference on Computing, Networking and Communications (ICNC).

[33] Bekele Y.Z., Choi Y.J. Scheduling for Machine Type Communications in LTE Systems. Proceedings of the 2018 International Conference on Information and Communication Technology Convergence (ICTC).

[34] Vilgelm M., Schiessl S., Al-Zubaidy H., Kellerer W., Gross J. On the reliability of LTE random access: Performance bounds for machine-to-machine burst resolution time. Proceedings of the 2018 IEEE International Conference on Communications (ICC).

[35] Lee J.Y., Noh H., Lee K., Choi J. Comparison of one-shot and handshaking systems for MTC in 5G. Proceedings of the 2018 IEEE 87th Vehicular Technology Conference (VTC Spring).

[36] Cheng R.G., Becvar Z., Huang Y.S., Bianchi G., Harwahyu R. (2019). Two-Phase Random Access Procedure for LTE-A Networks. IEEE Trans. Wirel. Commun..

[37] 3GPP (2014). Study on RAN Improvements for Machine-Type Communications.

[38] Akdeniz M.R., Liu Y., Samimi M.K., Sun S., Rangan S., Rappaport T.S., Erkip E. (2014). Millimeter wave channel modeling and cellular capacity evaluation. IEEE J. Sel. Areas Commun..

[39] Bekele Y.Z., June-Choi Y. Access Point Selection Using Reinforcement Learning in Dense Mobile Networks. Proceedings of the 2020 International Conference on Information Networking (ICOIN).

[40] NSNAM Ns-3: A Discrete-Event Network Simulator for Internet Systems, 2006–2020. https://www.nsnam.org/.

